# Allergen Exposure in Murine Neonates Promoted the Development of Asthmatic Lungs

**DOI:** 10.3390/biomedicines9060688

**Published:** 2021-06-18

**Authors:** Jeng-Chang Chen, Cheng-Chi Chan, Nai-Chun Ting, Ming-Ling Kuo

**Affiliations:** 1 Department of Surgery, Chang Gung Children’s Hospital, College of Medicine, Chang Gung University, Taoyuan 333, Taiwan; bx9619@cgmh.org.tw; 2 Department of Medicine, College of Medicine, Chang Gung University, Taoyuan 333, Taiwan; 3 Abnova Corporation, Taipei 114, Taiwan; ryanchan@abnova.com.tw; 4 Department of Microbiology and Immunology, Graduate Institute of Biomedical Sciences, College of Medicine, Chang Gung University, Taoyuan 333, Taiwan; 5 Graduate Institute of Clinical Medical Sciences, College of Medicine, Chang Gung University, Taoyuan 333, Taiwan; D0500503@cgu.edu.tw; 6 Department of Pediatrics, Division of Allergy, Asthma, and Rheumatology, Chang Gung Memorial Hospital, Taoyuan 333, Taiwan

**Keywords:** neonate, chemokine, cytokine, sensitization, ovalbumin, asthma, adjuvant

## Abstract

We previously demonstrated that fetal allergen exposure caused T-helper 2 (Th2) cell sensitization. Although neonates are immunologically more mature than fetuses, asthmatic lungs were reportedly mitigated by neonatal allergen administration, mechanically referring to regulatory T-cells and TGF-*β* signaling but lacking the immunological profiles after neonatal exposure. To reappraise the immunological outcome of neonatal allergen exposure, we injected adjuvant-free ovalbumin intraperitoneally into 2-day-old BALB/c neonates, followed by aerosolized ovalbumin inhalation in adulthood. Mice were examined for the immunological profiles specifically after neonatal exposures, lung function and histology (hematoxylin-eosin or periodic acid Schiff staining), and gene expressions of intrapulmonary cytokines (IL-4, IL-5, IL-13 and IFN-γ) and chemokines (CCL17, CCL22, CCL11 and CCL24). Neonatal ovalbumin exposure triggered Th2-skewed sensitization and ovalbumin-specific IgE production. Subsequent ovalbumin inhalation in adulthood boosted Th2 immunity and caused asthmatic lungs with structural and functional alterations of airways. Gender difference mainly involved airway hyperresponsiveness and resistance with greater female susceptibility to methacholine bronchospastic stimulation. In lungs, heightened chemoattractant gene expressions were only granted to neonatally ovalbumin-sensitized mice with aerosolized ovalbumin stress in adulthood, and paralleled by upregulated Th2 cytokine genes. Thus, aeroallergen stress in atopic individuals might upregulate the expression of intrapulmonary chemoattractants to recruit Th2 cells and eosinophils into the lungs, pathogenically linked to asthma development. Conclusively, murine neonates were sensitive to allergen exposures. Exposure events during neonatal stages were crucial to asthma predisposition in later life. These findings from a murine model point to allergen avoidance in neonatal life, possibly even very early in utero, as the best prospect of primary asthma prevention.

## 1. Introduction

According to Medawar, antigen exposure before full immune development during fetal stage might elicit tolerance to this specific antigen [1]. Such a concept, termed as “actively acquired tolerance”, has fascinated many researchers and attracted a number of laboratory work to replicate immune tolerance. Armed with Medawar’s knowledge, we had never attempted to prenatally abolish or diminish allergic responses in a murine model. However, artificial fetal allergen contact turned out to be an event of allergen-specific T-helper 2 (Th2) cell sensitization [2]. Prenatal imprinting of Th2 sensitization to allergens basically stood in sharp contrast to allogeneic tolerance induction following fetal exposure to allogeneic marrows [3]. In fact, fetal exposure to foreign antigens in animal studies over the past decades did not consistently induce immune tolerance as first envisaged by Medawar, but rather could be tolerogenic or immunogenic, depending upon the type or nature of antigens introduced [4].

Although neonates are ontogenically and immunologically more mature than fetuses, earlier studies reported immune tolerance or unresponsiveness following neonatal exposure to foreign antigens [5,6,7]. Contrarily, later studies disclosed that murine neonatal T cells were competent to mount balanced Th1/Th2 [8,9] or Th2-skewed [10,11] responses. Notably, neonatal tolerance was deemed to be Th2-skewed immune deviation rather than the induction of T-cell unresponsiveness [12]. Similar to what we observed in murine fetuses, the dose and type of antigens given to murine neonates might determine the immunological outcome [8,12,13,14]. Although epidemiological studies attributed allergen sensitization to early life exposure in humans [15], several murine studies revealed that neonatal allergen exposure lessened allergic airway responses [16,17]. These conflicting observations prompted us to reappraise the immunological consequences of neonatal exposure to allergens, and investigate its influences on asthma development later in life. Due to the effects of gender on asthma presentations in humans [18,19] and murine models [20], the data of female and male subjects were analyzed separately in this study. It was found that adjuvant-free ovalbumin (OVA) could trigger Th2-skewed atopy in murine neonates regardless of the gender. In neonatally OVA-sensitized female or male mice, repetitive allergen inhalation later in life not only boosted Th2 immune responses but also upregulated the expressions of intrapulmonary chemoattractants for Th2-cell and eosinophil recruitment into the lungs, pathogenically relevant to asthma development with structural and functional alterations of airways.

## 2. Materials and Methods

### 2.1. Animals

Four- to five-week-old male and female BALB/c mice were purchased from National Laboratory Animal Center (Taipei, Taiwan). For breeding, six- to eight-week-old female mice were caged with males in the afternoon and checked for vaginal plugs the following morning. The care and handling of mice in all experimental protocols were approved by the Institutional Animal Care Committee of Chang Gung University (CGU-106-209; 30/03/18) and complied with the Guidelines for the Care and Use of Laboratory Animals.

### 2.2. OVA Exposure and Challenge in Mice

**Figure 1:**
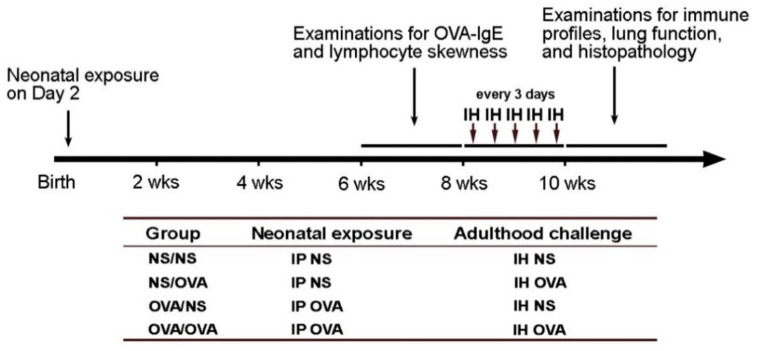
Schematic of experimental design for OVA exposure and challenge in mice, and grouping. IP, intraperitoneal injection; IH, inhalation; NS, normal saline; OVA, ovalbumin.

Newborn mice (2-day-old) were exposed to 50 μg adjuvant-free OVA (grade V, Sigma-Aldrich, St. Louis, MO, USA) in 50 μL of normal saline (NS) by intraperitoneal injection. Control mice were injected with NS alone. Six to eight weeks after OVA administration and before aerosolized OVA challenge, blood samples were collected under anesthesia from the retro-orbital venous plexus using heparinized capillary tubes. At the age of 8–10 weeks, NS and OVA-exposed mice were challenged by inhalation of 2% aerosolized OVA or NS for 20 min using an atomizer (Pulmo-aide DeVilbiss Health Care, Port Washington, NY, USA) every three days for five times (Figure 1).

### 2.3. Measurement of Lung Function

Airway hyperresponsiveness (AHR) was measured in unrestrained mice by whole-body plethysmograph (Buxco Electronics Inc., Wilmington, NC, USA) under methacholine (Sigma-Aldrich) stimulation. Following acclimation and saline inhalation for 3 min, conscious mice were exposed to aerosolized methacholine (10, 20, 30, and 40 mg/mL, respectively) for 3 min. The changes of enhanced pause (Penh) were recorded over 3 min for each methacholine dose and analyzed by BioSystem XA software (Buxco Electronics Inc.). 

For dynamic airway resistance, measurements were performed via a Buxco FinePointe RC system (Buxco Electronics, Inc.). Mice were anesthetized by intraperitoneal injection (10 mL/kg) of 6.67 mg/kg Zoletil 50 (Virbac, Carros cedes, France) and 3.89 mg/kg xylazine hydrochloride (Bayer, Leverkusen, Germany), then tracheostomized and immediately cannulated with an 18G catheter for mechanical ventilation. Aerosolized methacholine (3.3, 10, and 30 mg/mL, respectively) was administered via a nebulizer aerosol system for 30 s. Responses in total resistance were analyzed over a 3-min period after the last puff. Airway resistance was computed from the tracheal pressure and airflow signals. Airway resistance baseline was recovered for 1 min before the administration of subsequent methacholine stimulation.

### 2.4. Eosinophil Enumeration in Bronchoalveolar Lavage Fluid (BALF) and Lung Histology

After AHR measurement, the tracheas of mice in different groups were intubated, and the lungs were lavaged three times with 1 ml phosphate buffer saline (PBS) supplemented with 0.1 mM EDTA. Cells in BALF were collected after centrifugation (Shandon Cytospin 4; ThermoFisher Scientific, Waltham, MA, USA), and stained with Wright-Giemsa staining solution. Based on the staining morphology of bilobed nuclei and eosinophilic cytoplasmic granules under a light microscope, the eosinophil percentage as the severity of eosinophilia was determined by the ratio of eosinophils enumerated to at least 200 cell counts collected from BALF.

Lung tissues (without lavage) were obtained and fixed with 4% formaldehyde at 4 °C for histological analyses. After dehydration, the tissues were embedded with paraffin and sliced into 4-μm sections. Then, paraffin tissue sections were subjected to hematoxylin and eosin (H&E) or periodic acid Schiff (PAS; Sigma) staining for histological examinations. Leukocyte infiltration (as inflammation index) and goblet cell hyperplasia (as mucin deposition) were analyzed and quantified by ImageJ software (V1.51, NIH, USA) with Threshold_Colour plugin. In each tissue section, five 100-μm^2^ areas were selected for analyses. The integrated intensity of each area was determined by HSB assay (Hue: 180–255, Saturation: 62–255, Brightness: 0–255 for H&E; Hue: 170–195, Saturation: 80–255, Brightness: 0–255 for PAS). Their mean intensity was recorded as individual histological scores of inflammation index and mucin deposition.

### 2.5. Measurement of OVA-Specific IgE (OVA-IgE)

The levels of OVA-IgE in serum were measured by ELISA. Briefly, plates (Costar, Corning, NY, USA) were coated with 10 μg/mL OVA at 37 °C for 1 h. OVA-coated plates were incubated with diluted serum samples at 37 °C for 1 h. Subsequently, the biotinylated rat anti-mouse IgE monoclonal antibodies (R35-118; BD PharMingen, San Diego, CA, USA) and streptavidin conjugated-horseradish peroxidase (HRP, BD PharMingen) were added to the plates. Finally, plates were incubated with substrate solution (TMB, BD Biosciences, Franklin Lakes, NJ, USA) for 20 min, and the reaction was stopped with 2N H_2_SO_4_. Absorbance was measured by ELISA reader at 450 nm (SpectraMax M2, Molecular Devices, San Jose, CA, USA).

### 2.6. Cell Culture and Cytokine Assays

Spleens were obtained and gently dissociated in sterile PBS. Splenocytes were treated with red blood cell (RBC) lysing buffer for depleting RBCs and passed through a 70 μm cell strainer for the preparation of single-cell suspensions. Cells (5 × 10^6^ cells/mL) were cultured in RPMI 1640 medium (Gibco, ThermoFisher Scientific) containing 10% heat-inactivated fetal bovine serum (FBS, Gibco) and 1% penicillin/streptomycin (Gibco) with or without 100 μg/mL OVA in 24-well plates. Culture supernatants were harvested on day 6. The concentrations of interleukin (IL)-4, IL-13, interferon gamma (IFN-γ) (DuoSet ELISA kit; R&D Systems, Minneapolis, MN, USA), and IL-5 (BD OptEIA set; BD Pharmingen) were determined by ELISA according to the manufacturers’ instructions.

### 2.7. RNA Isolation and Quantitative PCR

Total RNA was extracted from homogenized lung tissues using TRIzol reagent (Invitrogen, Life Technologies) and treated with DNase I (Fermentas, ThermoFisher Scientific) for removing template DNA. The cDNA was generated using random primers and M-MLV reverse transcriptase (Invitrogen, ThermoFisher Scientific). The sequences of specific primers for quantitative PCR (qPCR) of *IL-4*, *IL-5*, *IL-13*, *IFN-γ*, *CCL11*, *CCL17*, *CCL22*, *CCL24*, *Gob5*, *MUC5AC*, and *β-actin* were described previously [21,22,23]. Diluted cDNA samples were amplified using SYBR Green/ROX qPCR Master Mix (Fermentas) in a CFX Connect real-time PCR detection system (Bio-Rad, Hercules, CA, USA). The cycling conditions were 50 °C for 2 min, 95 °C for 10 min, and 40 cycles of 95 °C for 15 s and 60 °C for 1 min. The relative expression of each gene was calculated by normalizing the level to the expression of housekeeping *β-actin* gene.

### 2.8. Statistical Analysis

All bar data were presented as 95% confidence intervals for the means. The equality of means was examined by Student’s *t*-test between two independent or paired groups, or by one-way analysis of variance (ANOVA) among three or more groups with post hoc multiple comparisons using Bonferroni method. Differences were regarded as significant in all tests at *p* < 0.05.

## 3. Results

### 3.1. Immunological Consequences of Neonatal Exposure to OVA

**Figure 2:**
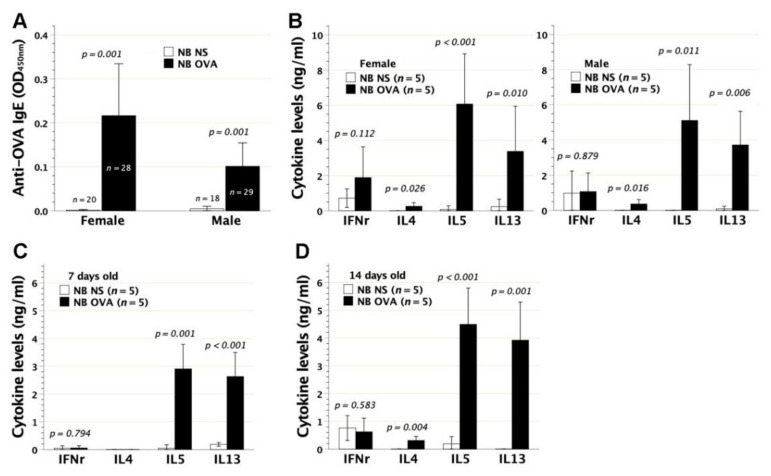
The generation of OVA-IgE and Th2 immune deviation following neonatal OVA exposure. BALB/c newborns (NB) were intraperitoneally exposed to adjuvant-free OVA of 50 μg at 2 days old (NB OVA). Serum OVA-IgE was measured by ELISA and lymphocyte skewness was in vitro examined under OVA stimulation. (**A**) At 6–8 weeks of age, both female and male recipients generated significant levels of OVA-IgE, as compared with control neonates receiving saline injection (NB NS). Serum OVA-IgE of NB OVA did not differ in mean titer between females and male (*p* = 0.076) (**B**) Lymphocyte polarization was examined by IFN-γ, IL-4, IL-5, and IL-13 secretions in cell cultures under OVA stimulation. NB OVA compared favorably in the levels of IL-4, IL-5, and IL-13 but not IFN-γ with NB NS, indicating a Th2-skewed phenotype. (**C**) Th2-skewed lymphocytes were characterized by IL-5 and IL-13 secretions at 7 days old, and (**D**) by IL-4, IL-5, and IL-13 secretions at 14 days old. Days 7 and 14 cytokine data were generated from subjects without specifying the gender due to the difficulty in visually telling female from male animals at this stage.

To evaluate the immunological consequences of neonatal allergen exposure, we subjected BALB/c neonates to intraperitoneal injection of adjuvant-free OVA on day 2 after birth. At 6–8 weeks of age, serum OVA-IgE, measured by ELISA, was significantly detected in both female and male recipients (Figure 2A). As a result, neonatal contact with adjuvant-free OVA elicited a sensitization event regardless of their gender. Despite a visually higher mean titer of serum OVA-IgE in females than in males, the difference did not reach statistical significance. Furthermore, splenic lymphocytes of murine recipients were examined for their immune deviation under OVA stimulation in an in vitro culture system. In culture supernatants, recipients’ lymphocytes compared favorably in the generation of IL-4, IL-5, and IL-13 with those of saline controls, whereas it made no difference in the levels of IFN-γ secreted (Figure 2B). Female and male recipients shared an analogous pattern of IFN-γ, IL-4, IL-5, and IL-13 secretions by lymphocytes. Th2 cytokines of IL-4, IL-5, and IL-13 were barely detected in lymphocyte cultures supplemented with third-party stimulators of bovine serum albumin in both experimental and control mice (data not shown). Thus, neonatal OVA exposure on day 2 after birth caused Th2 skewness of recipients’ lymphocytes. Among the Th2 cytokines secreted, IL-5 and IL-13 swiftly showed up by the age of 7 days (Figure 2C), whereas IL-4 was additionally secreted by the age of 14 days (Figure 2D).

### 3.2. Lung Parameters in Mice Subjected to Neonatal OVA Exposure

**Figure 3:**
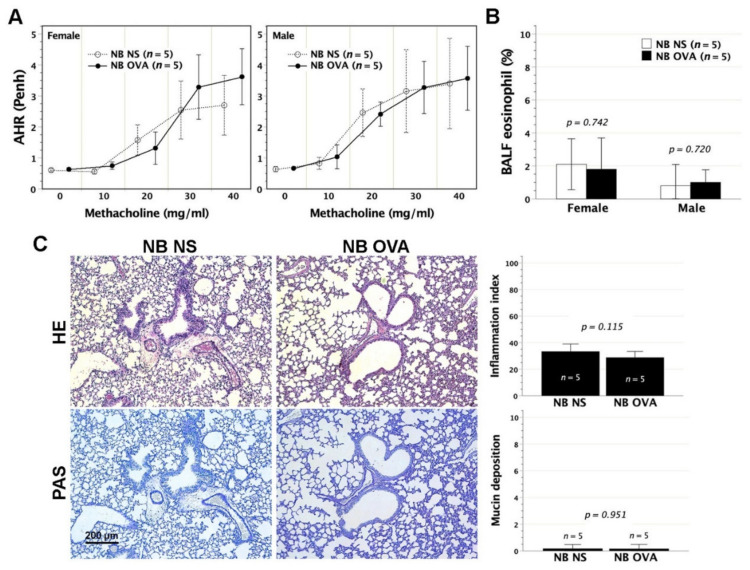
Pulmonary function and histology following neonatal OVA exposure. Following neonatal exposure to OVA of 50 μg (NB OVA), mice at 2 months old were subjected to pulmonary function test of AHR under aerosolized methacholine stimulation using whole-body plethysmograph. (**A**) In recipients regardless of gender, AHR expressed as Penh did not differ between NB OVA and saline controls (NB NS) upon bronchospastic stimuli at any aerosolized methacholine dose. (**B**) As to eosinophil percentage in BALF, no significant difference was observed between NB OVA and NB NS. (**C**) Representative images were taken from females with neonatal OVA exposure. There were no significant histological alterations in terms of leukocyte infiltration and goblet cell hyperplasia/mucin deposition, which were common to males. HE: hematoxylin and eosin; PAS: periodic acid Schiff.

Neonatally OVA-exposed mice were evaluated for their lung parameters including pulmonary function test, eosinophil fractions in BALF and histopathological alterations. Pulmonary function test was examined in response to escalating doses of aerosolized methacholine using non-invasive whole-body plethysmography. The Penh values were dose-responsive to methacholine stimulation in both female and male recipients, but did not differ from those of their individual saline controls (Figure 3A). Thus, neonatal OVA exposure did not heighten airway hyperresponsiveness to methacholine stimulation later in adult life. We further examined the lung histology and eosinophil fractions of BALF. Peritoneal exposure to OVA in neonates did not elicit inflammatory cell infiltration, nor did it cause the increment of eosinophil fraction in BALF (Figure 3B) and histologically structural alterations of goblet cell metaplasia/hyperplasia within the lungs (Figure 3C).

### 3.3. Heightened Recall Immune Responses and Hyperresponsive Airways in Neonatally OVA-Exposed Mice Following Aerosolized OVA Challenge in Adulthood

**Figure 4:**
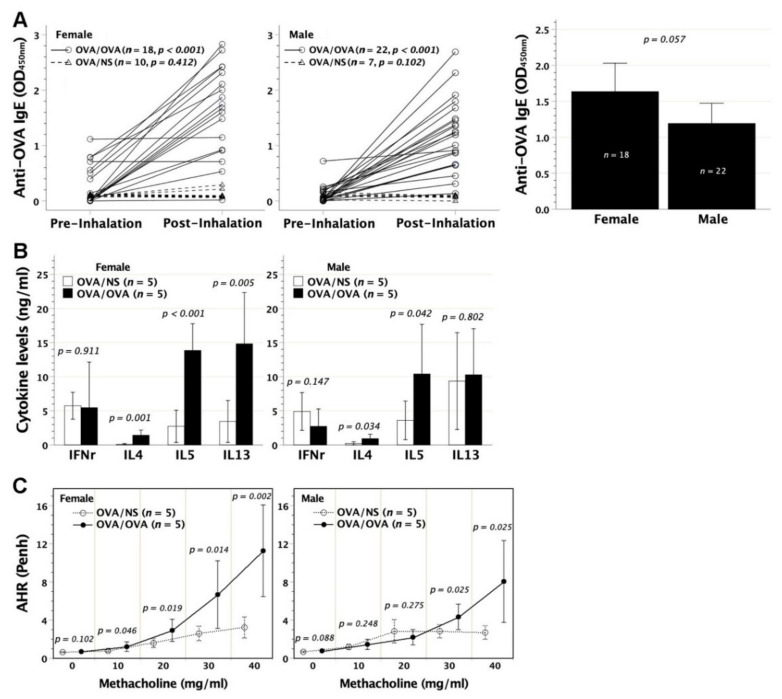
Immunological outcome and airway hyperresponsiveness in neonatally OVA-sensitized mice following airway OVA stress. BALB/c neonates were intraperitoneally sensitized to OVA at 2 days old, and further subjected to repetitive OVA inhalation at 8–10 weeks old (OVA/OVA). (**A**) Both female and male recipients showed significantly raised levels of serum OVA-IgE after OVA inhalation (paired comparison), as opposed to neonatally OVA-sensitized mice subjected to saline inhalation (OVA/NS). The mean titer of serum OVA-IgE after OVA inhalation in OVA/OVA made no difference between females and males (*p* = 0.057). (**B**) In cell culture under OVA stimulation, group OVA/OVA exhibited enhanced lymphocyte capacity for generating Th2 cytokines of IL-4, IL-5, and IL-13 in females, but only IL-4 and IL-5 in males, as compared with group OVA/NS. It made no difference in the generation of Th1 IFN-γ between groups OVA/OVA and OVA/NS. (**C**) AHR significantly increased upon bronchospastic stimuli at 10–40 mg/mL of aerosolized methacholine in females, but at 30–40 mg/mL in males.

To evaluate the influence of aerosolized OVA stress on the immunological profiles and bronchospastic responses of neonatally OVA-exposed mice, we subjected neonatally OVA-exposed recipients to repetitive OVA inhalation at 8–10 weeks of age. This treatment significantly boosted serum OVA-IgE levels in both female and male recipients (Figure 4A). There was no significant difference in mean titer of boosted serum OVA-IgE between females and males. Moreover, aerosolized OVA stress on neonatally OVA-exposed mice enhanced the secretion of IL-4 and IL-5 rather than IFN-*γ* in OVA-stimulated lymphocyte cultures regardless of gender. However, IL13 secretion was intensified only in female recipients (Figure 4B). OVA inhalation also endowed neonatally OVA-exposed mice with increased Penh in response to methacholine bronchospastic stimuli as compared with their counterpart saline controls (Figure 4C), indicating hyperresponsive airways following airway OVA challenge. Noticeably, females showed a more sensitive airway response to methacholine than males, evidenced by significant Penh increment beginning at the methacholine dose of 10 mg/mL in females as opposed to 30 mg/mL in males.

### 3.4. Immunological Consequences of Aerosolized OVA Exposure in Adulthood

**Figure 5:**
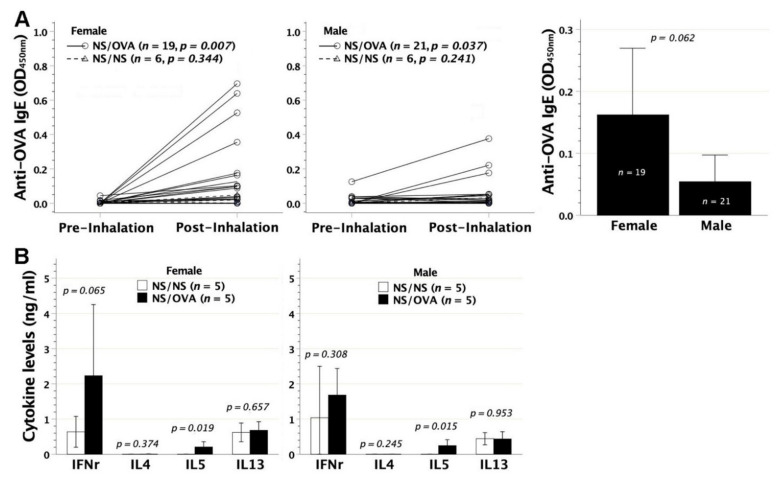
Immunological outcome of OVA inhalation in adulthood. Two-month-old BALB/c adults that had been given intraperitoneal saline injection at 2 days old were subjected to repetitive OVA inhalation every 3 days for a total of 5 times (NS/OVA). (**A**) OVA inhalation in both female and male recipients led to the secretion of serum OVA-IgE, as opposed to the recipients with neonatal saline injection followed by saline inhalation in adulthood (NS/NS). After OVA inhalation, the mean titer of serum OVA-IgE in NS/OVA did not differ between females and males (*p* = 0.062). (**B**) In lymphocyte culture under OVA stimulation, group NS/OVA regardless of gender compared favorably in the generation of IL-5 rather than IFN-*γ*, IL-4, and IL-13 with group NS/NS.

To examine the immunological outcome of aerosolized OVA stress in adulthood, we subjected neonatally saline-exposed mice to repetitive OVA inhalation at 8–10 weeks of age. OVA aerosol stress in adult mice neonatally manipulated by intraperitoneal saline injection could significantly induce the generation of serum OVA-IgE in sera (Figure 5A), which did not statistically differ between females and males. As to lymphocyte polarization, Th2 immune deviation was mainly featuring IL-5, but lacking IL-4 and IL-13 secretions (Figure 5B). Thus, repetitive OVA inhalation in murine adulthood remained immunogenic to skew lymphocytes towards the Th2 phenotype that was limited to IL-5 secretion.

### 3.5. Asthmatic Lungs in Neonatally OVA-Exposed Mice Subjected to Aerosolized OVA Stress

**Figure 6:**
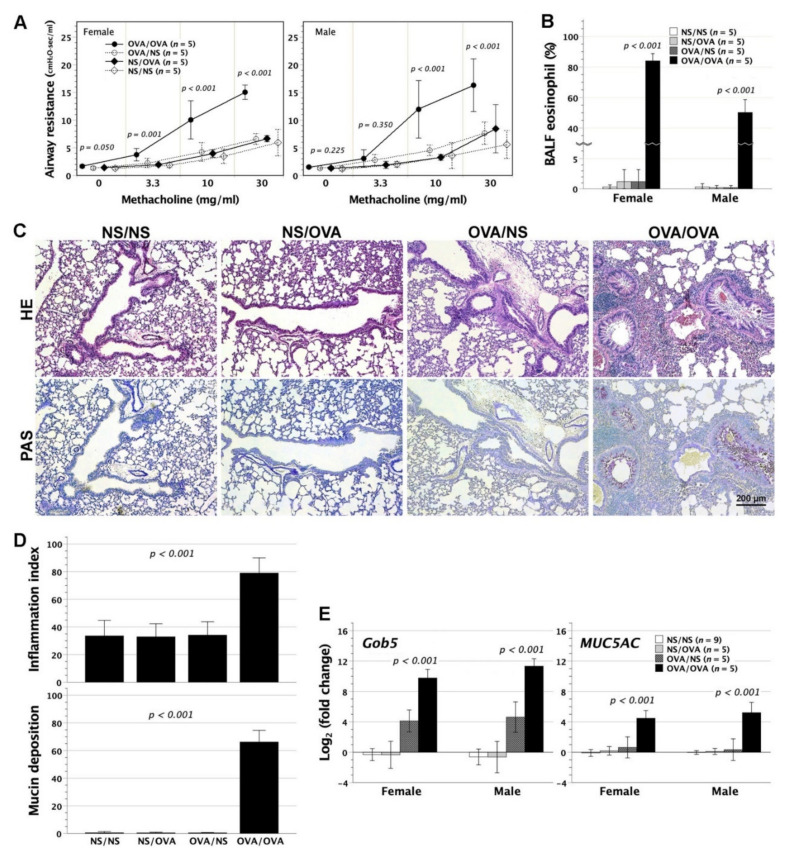
Airway resistance, histological alteration, and mucin-related gene expression in neonatally OVA-sensitized mice with aerosolized OVA stress in adulthood. Two-day-old BALB/c neonates underwent intraperitoneal OVA injection, and then received OVA inhalation 5 times at 2 months old (OVA/OVA). (**A**) Airway resistance to methacholine stimuli was measured in tracheostomized animals, using a FinePointe^TM^ RC system. In group OVA/OVA, airway resistance notably rose in females as methacholine bronchospastic stimuli increased from 3.3 to 30 mg/mL, but in males from 10 to 30 mg/mL, as compared with that of groups OVA/NS, NS/NS, and NS/OVA. There was no significant difference in airway resistance among groups OVA/NS, NS/NS, and NS/OVA. (**B**) Group OVA/OVA compared favorably in BALF eosinophil percentage with groups OVA/NS, NS/NS and NS/OVA. (**C**) Histological examination revealed peribronchial and perivascular inflammation (HE staining), and extensive goblet cell hyperplasia of airways with luminal mucin deposition (PAS staining) in the lungs of group OVA/OVA. Representative images were taken from females. Females and males had common patterns of histological findings. (**D**) The severity of peribronchial/perivascular inflammation and mucin deposition on captured images was quantified by image analysis software. Group OVA/OVA compared favorably in integrated intensity of inflammation and mucin deposition with groups OVA/NS, NS/NS, and NS/OVA. (**E**) The expression of *Gob5* and *MUC5AC* genes was determined by quantitative PCR and normalized to β-actin expression. The fold change was the expression ratio as the normalized expression level of a subject divided by the mean of normalized expression levels in group NS/NS. Group OVA/OVA compared favorably in *Gob5* and *MUC5AC* expression with groups OVA/NS, NS/NS, and NS/OVA. There was no significant difference in *Gob5* and *MUC5AC* expression among groups OVA/NS, NS/NS, and NS/OVA by post hoc multiple comparisons using Bonferroni method. OVA/NS: neonatal OVA exposure and adulthood saline inhalation; NS/NS: neonatal saline exposure and adulthood saline inhalation; NS/OVA: neonatal saline exposure and adulthood OVA inhalation.

Following aerosolized OVA stress, the neonatally OVA-exposed mice were scrutinized for the development of asthmatic lungs, including airway resistance, BALF eosinophil fractions, and histopathological alterations. Airway resistance to methacholine stimuli was measured in tracheostomized animals, using a FinePointeTM RC system. In neonatally OVA-sensitized BALB/c mice, aerosolized OVA challenge led to increased airway resistance under methacholine bronchospastic stimuli (Figure 6A). Of note, females showed a more sensitive bronchospastic response of airways than males, evidenced by significant increment of airway resistance beginning at the methacholine dose of 3.3 mg/mL in females as opposed to 10 mg/mL in males. Aerosolized OVA stress also provoked remarkable eosinophilia in BALF (Figure 6B). On histopathologic examinations, OVA inhalation in neonatally OVA-sensitized subjects induced massive infiltration of inflammatory cells in the peribronchial and perivascular area of lungs, and extensive goblet cell metaplasia/hyperplasia in association with copious mucin secretion mixed with inflammatory cells in airways (Figure 6C,D).

Airway structural and functional alterations were further verified by *Gob5* and *MUC5AC* gene expression in lungs. Gob5, as a calcium-activated chloride channel, involves in the regulation of mucus production and/or secretion and is the key molecule in murine asthma induction [24,25]. Regulated by Gob5 [24], MUC5AC are restricted to goblet cells and upregulated during allergic airway inflammation [26]. In keeping with the histopathological findings, both *Gob5* and *MUC5AC* genes were highly upregulated, 2^10–12^ and 2^4–6^ folds respectively, in the lungs of neonatally OVA-sensitized mice which were further subjected to aerosolized OVA stress (Figure 6E). There was an upward trend of *Gob5* gene expression in neonatally OVA-sensitized mice with saline inhalation as opposed to neonatally saline-exposed mice (NS/NS and NS/OVA). However, the difference could not reach statistical significance by multiple comparisons. Thus, aerosolized OVA stress in neonatally OVA-sensitized mice triggered asthmatic lungs with *Gob5*/*MUC5AC* gene upregulation, airway eosinophilia, and structural alterations in association with marked increment of airway resistance to bronchospastic stimuli.

It was worth mentioning that adulthood OVA inhalation in neonatally saline-injected mice did not cause any substantial alterations of airway resistance, BALF eosinophil fractions, lung histopathology, and pulmonary *Gob5*/*MUC5AC* gene expression, indicating the crucial role of prior neonatal OVA exposure in the development of asthmatic lungs.

### 3.6. Cytokine and Chemokine Gene Expression in the Lungs

**Figure 7:**
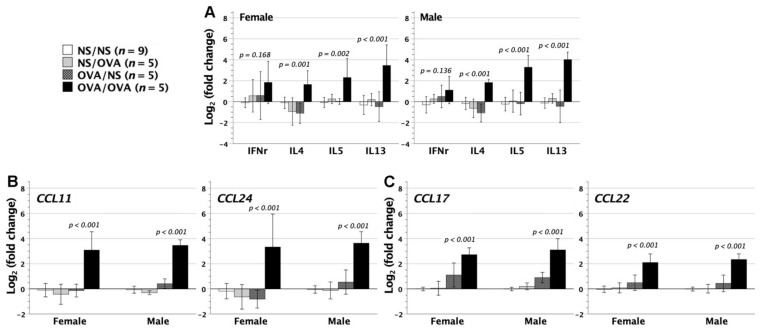
Gene expression of cytokines and chemokines in the lungs. Neonatally OVA-sensitized mice with adulthood aerosolized OVA stress (OVA/OVA) were examined for cytokine and chemokine gene expression of the lungs. Control mice were neonatally saline-exposed mice with adulthood saline inhalation (NS/NS). (**A**) Regardless of gender, group OVA/OVA had significantly upregulated expression of IL-4, IL-5, and IL-13 for 2^2–4^ folds as compared with group NS/NS, whereas IFN-γ gene expression did not differ between groups OVA/OVA and NS/NS. (**B**,**C**) Likewise, gene expression of chemokines CCL11, CCL24, CCL17, and CCL22 in the lungs showed 2^2–3^-fold increase in group OVA/OVA, as opposed to group NS/NS. Neither group NS/OVA nor group OVA/NS had any significant changes of cytokines or chemokine gene expression when compared with group NS/NS.

In mice with neonatal saline or OVA exposure followed by saline or OVA inhalation, we further quantified the gene expressions of cytokines IFN-*γ*, IL-4, IL-5, and IL-13 in their lungs. Mice with neonatal saline exposure and adulthood saline inhalation were used as the controls. There was no significant difference in IFN-*γ* gene expression among the four groups, whereas mice with neonatal OVA exposure followed by adulthood OVA inhalation exhibited the upregulation of IL-4, IL-5, and IL-13 Th2 cytokine genes in the lungs, as opposed to the other three groups (Figure 7A).

Given that neonatally OVA-exposed mice exhibited severe inflammation of lungs after aerosolized OVA inhalation, we further analyzed the relevant chemokine gene expression in the lungs. CCL11 and CCL24 have important implications for allergic responses [27]. They are produced locally by epithelial, mesenchymal, or endothelial cells and crucial to eosinophil recruitment and priming for mediator secretion once they reach airways [28,29]. OVA inhalation significantly upregulated both *CCL11* and *CCL24* expression in the lungs of neonatally OVA-exposed mice (Figure 7B), compatible with the presence of BALF eosinophilia (Figure 6B).

CCL17 and CCL22 chemokines, mainly from dendritic cells [30], are involved in T-cell chemotaxis, especially Th2 cells by binding to the chemokine receptor CCR4 [31]. They play an important role in the induction of various inflammatory responses, particularly allergic diseases [32]. In neonatally OVA-exposed mice, further aerosolized OVA stress caused the upregulation of *CCL17* and *CCL22* gene expression in the lungs (Figure 7C). The upregulation of intrapulmonary CCL17 and CCL22 chemokines might recruit CD4^+^ Th2 inflammatory cells into the lungs of neonatally OVA-exposed mice, in keeping with the upregulation of intrapulmonary Th2 cytokine genes (Figure 7A). It indicated the crucial role of CCL17 and CCL22 chemokines in the pathogenesis of asthmatic lungs.

For all cytokines and chemokines examined, female and male mice showed similar patterns of gene expression.

## 4. Discussion

The incidence of asthma and other allergic diseases continues to increase during childhood and even become a major public health issue in industrialized and developing countries [33]. Atopy, characterized by Th2 cell-driven immune responses specific to allergens, is considered to be the important risk factor for the development of allergic diseases [34,35]. Thus, the timing of atopy initiation is crucial to allergy prevention in public health. There is a large body of evidence showing that atopy is initiated by early life events, especially in utero exposures [36,37]. In our previous study, allergen contact in pre-immune murine fetuses triggered Th2-skewed atopy, which promoted asthma development in the case of aerosolized allergen stress in postnatal life [2]. It supported a link of atopy development to prenatal allergen contact, in keeping with the clinical evidence for fetal sensitization by maternally derived allergens [38] including the detection of transplacental allergen transfer [39,40], and the presence of allergen-specific IgE and T-cell memory in cord blood [35]. In this study, OVA exposure in 2-day-old murine neonates also elicited sensitization to rapidly skew neonatal immune responses towards Th2 phenotypes within one week. Subsequent aeroallergen inhalation repeatedly in adulthood led to asthmatic lungs with functional and structural alterations of airways. Without prior neonatal OVA exposure, repetitive adulthood OVA inhalation was unable to induce asthma development in terms of the absence from any substantial alterations of AHR, airway resistance, lung histology, as well as intrapulmonary expressions of relevant genes (*Gob5*/*MUC5AC*, cytokines, and chemokines), but remained sufficient to elicit the generation of serum OVA-IgE and IL-5 secreting Th2 cells. Thus, adulthood OVA inhalation alone was immunogenic but not asthmogenic. Taken together, allergen contact in neonatal life essentially caused sensitization to set up Th2-skewed immune milieu, which was crucial to the induction of asthmatic lungs by later life aeroallergen stress. These findings depicted the disease process of asthma as neonatal allergen sensitization followed by aerosolized allergen stress repeatedly later in life. Neither alone was able to cause asthmatic lungs.

In a birth cohort study, early life exposure to inhalant or food allergens could be linked to a state of allergic sensitization [15]. Moreover, aeroallergen exposure in early childhood was prospectively demonstrated to determine the subsequent development of asthma [41]. Several epidemiologic investigations disclosed that the season of birth had an influence on the subsequent development of seasonal pollen allergy, suggesting that there might be a period of time after birth in which the neonatal or infant’s immune system is particularly susceptible to pollen sensitization [42]. Given that seasonal pollen allergy was usually diagnosed in school-age children [42], and epidemiologically correlated with asthma development even later in life [43], it would take years for pollen-sensitized neonates or infants to develop allergy or asthma through repetitive pollen rechallenge in a seasonal fashion [42]. Such a temporal sequence of disease process could be reflected by the schematic of experimental design for asthmatic lung induction in this murine study. Our results supported that the seeds of asthmatic diathesis were sown early in life. This knowledge justified allergen avoidance in early life for asthma prevention.

Although we showed Th2-skewed sensitization following intraperitoneal exposure to OVA in murine neonates, there is no shortage of studies reporting that allergic airway responses could be attenuated or prevented by intraperitoneal [44], intranasal [45], or oral [16,17] administration of allergens in murine neonates. In sharp contrast to neonatal sensitization in this study, neonatal injection of high-dose adjuvant-free OVA (1 mg) in mice was reported to downregulate Th2 immune milieu, which failed to support asthmatic lung induction by aerosolized OVA inhalation [44]. Notably, these studies did not scrutinize cellular and humoral immunity to allergens after neonatal exposures, but instead focused on the final outcome of asthma induction after subsequent allergen sensitization and challenge in adulthood. The attenuation or prevention of asthma development was indeed observed but attributed to peripheral mechanisms of regulatory T-cells [16,17,45] and TGF-*β* signaling [16]. However, it remained unknown whether or not neonatal recipients had been rendered tolerant to allergens after neonatal exposures in their studies. Given that tolerization with lessening of AHR and inflammation might happen to mice following long-term allergen challenge [46,47], and was highly relevant to regulatory T-cells expressing membrane-bound TGF-*β* and FOXP3 [48], it was conceivable that the mitigation of allergic airway responses in these studies resulted from peripheral tolerance induced by long-term allergen challenge in adulthood rather than central deletional tolerance following neonatal allergen exposure.

Murine models of allergic asthma have been established by different sensitization and challenge protocols mainly in adulthood [47] despite early life events being reported to influence the development of allergic airway diseases [42]. The typical protocols to sensitize an adult immune system demanded repetitive systemic administration of allergens in the presence of an adjuvant [46,47]. Adjuvants could increase immunogenic efficacy of allergens, but might have significant influences on immune deviation [8]. Complete Freund’s adjuvants (CFA) predisposed immune responses to Th1 phenotypes [8,44], whereas aluminum hydroxide (AlOH_3_) or incomplete Freund’s adjuvants (IFA) promoted Th2 phenotypes [8,44,49]. Thus, the supplement of adjuvants might modify or alter the immunological process or outcome that was going to be modelled in animals exposed to allergens. Adjuvant-free protocols have also been adopted in murine adults [50,51], but demanded a larger number of exposures to attain appropriate sensitization. However, it was not the case in murine fetuses [2] or neonates, wherein a single-dose injection of adjuvant-free OVA sufficed to elicit sensitization. As a result, murine immune system in perinatal life, despite being relatively naïve and immature as opposed to adulthood, is more susceptible to allergens. Without the requirement of adjuvants for potentiating immunogenicity of allergens in murine fetuses or neonates, the unwanted influences of adjuvants on immune responses can be avoid. It makes a fetal or neonatal approach ideal for the setup of an asthma model in mice. Being less technically challenging than fetal injection, neonatal injection may be the superior strategy for initiating Th2 sensitization to facilitate asthma development by subsequent allergen inhalation later on in life. However, this neonatal model still suffers from the limitation that intraperitoneal injection is not the natural route of allergen exposure.

Gender difference, specifically termed as sexual dimorphism, has been a well-recognized phenomenon in adult mice subjected to asthma induction. Differences might encompass immunological [20,52,53,54], inflammatory [20,53,54,55], airway [54], or histological [53] parameters, varying according to the protocols used for asthma induction. Generally, female mice exhibited greater susceptibility to asthma induction than their male counterparts, characterized by a preponderance of experimental asthma parameters with remarkable alterations. In this murine model of neonatally-induced asthma, sexual dimorphism was mainly reflected by AHR and airway resistance with greater female susceptibility to methacholine bronchospastic stimulation. The potential effects of gender on murine asthmatic lungs essentially parallel the epidemiology of human asthma with greater susceptibility and severity in women [18,19], which was reportedly relevant to sex hormones [56,57] and gender difference in immune function itself [58,59]. Gender difference might be also the reason why aerosolized OVA stress in adulthood intensified IL-13 secretion only in neonatally OVA-sensitized female recipients of this study.

Asthmatic lungs were characterized by airway eosinophilia, hyperresponsiveness, and remodeling [60]. These disease features were causally driven by Th2 cytokines of IL-4, IL-5, or IL-13, as demonstrated by transgenic studies in mice [61,62,63]. The quantitative measurement of *IFN-γ*, *IL-4*, *IL-5*, and *IL-13* gene expression in the lungs further revealed that neither neonatal OVA injection nor adulthood OVA inhalation alone sufficed to upregulate the intrapulmonary Th2 cytokine expression. In neonatally OVA-exposed mice, it was not until adulthood OVA inhalation that intrapulmonary Th2 cytokine expression was upregulated. Of note, the upregulation of intrapulmonary Th2 cytokine expression was paralleled by not only the presence of remodeling and increased resistance in airways, but also the upregulation of intrapulmonary CCL17, CCL22, CCL11, and CCL24 chemokine gene expression. As we know, CCL17 and CCL22 might contribute to Th2 cell chemotaxis and airway inflammation [64], whereas CCL11 and CCL24 were potent chemoattractants for eosinophils [28,29]. Taken together, airway OVA stress in neonatally OVA-exposed mice might upregulate intrapulmonary chemokines of CCL17, CCL22, CCL11, and CCL24 to recruit Th2 CD4^+^ cells and eosinophils into the lungs, where the release of Th2 cytokines and relevant mediators led to eosinophilic inflammation and structural and functional alterations of airways.

## 5. Conclusions

Although murine neonates have been historically regarded as immunologically immature, reappraisal of neonatal allergen exposure demonstrated an event of sensitization that led to Th2 atopy, analogous to in utero sensitization [2]. As opposed to adulthood sensitization that demanded multiple injections of adjuvant-supplemented OVA [46,47], sensitization during intrauterine [2] and neonatal life only necessitated a single-dose injection of adjuvant-free OVA. It implied that murine fetuses and neonates were more sensitive to allergen exposures. In an individual with Th2 atopy initiated in early life, aeroallergen airway stress boosted Th2 immunity and gave rise to asthmatic lungs with structural and functional alterations of airways, and the upregulated gene expression of intrapulmonary CCL17, CCL22, CCL11, and CCL24 chemoattractants as well as Th2 cytokines. Thus, aeroallergen stress in atopic individuals might upregulate the expression of intrapulmonary chemoattractants to recruit Th2 cells and eosinophils into the lungs, pathogenically relevant to asthma development. Conclusively, exposure events during in utero and neonatal stages played an important role in modulating asthma predisposition in later life. Our results point to allergen avoidance in neonatal life, possibly even very early in utero, as the best prospect of primary asthma prevention.

## Data Availability

All data are contained in this article.
